# In vivo virulence of MHC-adapted AIDS virus serially-passaged through MHC-mismatched hosts

**DOI:** 10.1371/journal.ppat.1006638

**Published:** 2017-09-20

**Authors:** Sayuri Seki, Takushi Nomura, Masako Nishizawa, Hiroyuki Yamamoto, Hiroshi Ishii, Saori Matsuoka, Teiichiro Shiino, Hironori Sato, Kazuta Mizuta, Hiromi Sakawaki, Tomoyuki Miura, Taeko K. Naruse, Akinori Kimura, Tetsuro Matano

**Affiliations:** 1 AIDS Research Center, National Institute of Infectious Diseases, Tokyo, Japan; 2 Center for AIDS Research, Kumamoto University, Tokyo, Japan; 3 Pathogen Genomics Center, National Institute of Infectious Diseases, Tokyo, Japan; 4 Institute for Frontier Life and Medical Sciences, Kyoto University, Kyoto, Japan; 5 Medical Research Institute, Tokyo Medical and Dental University, Tokyo, Japan; 6 The Institute of Medical Science, The University of Tokyo, Tokyo, Japan; Emory University, UNITED STATES

## Abstract

CD8^+^ T-cell responses exert strong suppressive pressure on HIV replication and select for viral escape mutations. Some of these major histocompatibility complex class I (MHC-I)-associated mutations result in reduction of *in vitro* viral replicative capacity. While these mutations can revert after viral transmission to MHC-I-disparate hosts, recent studies have suggested that these MHC-I-associated mutations accumulate in populations and make viruses less pathogenic *in vitro*. Here, we directly show an increase in the *in vivo* virulence of an MHC-I-adapted virus serially-passaged through MHC-I-mismatched hosts in a macaque AIDS model despite a reduction in *in vitro* viral fitness. The first passage simian immunodeficiency virus (1pSIV) obtained 1 year after SIVmac239 infection in a macaque possessing a protective MHC-I haplotype *90-120-Ia* was transmitted into *90-120-Ia*^-^ macaques, whose plasma 1 year post-infection was transmitted into other *90-120-Ia*^-^ macaques to obtain the third passage SIV (3pSIV). Most of the *90-120-Ia*-associated mutations selected in 1pSIV did not revert even in 3pSIV. 3pSIV showed lower *in vitro* viral fitness but induced persistent viremia in *90-120-Ia*^-^ macaques. Remarkably, 3pSIV infection in *90-120-Ia*^+^ macaques resulted in significantly higher viral loads and reduced survival compared to wild-type SIVmac239. These results indicate that MHC-I-adapted SIVs serially-transmitted through MHC-I-mismatched hosts can have higher virulence in MHC-I-matched hosts despite their lower *in vitro* viral fitness. This study suggests that multiply-passaged HIVs could result in loss of HIV-specific CD8^+^ T cell responses in human populations and the *in vivo* pathogenic potential of these escaped viruses may be enhanced.

## Author summary

CD8^+^ T-cell responses exert considerable control over replication of HIV and select for viral escape mutations. Recent studies have suggested that these major histocompatibility complex class I (MHC-I)-associated mutations accumulate in populations and make viruses less pathogenic *in vitro*. Other studies have shown that some of these escape mutations can revert after passage to MHC-I-disparate hosts. In an attempt to reconcile these apparently conflicting results, we serially passaged a virus isolate through MHC-I-mismatched hosts in the macaque AIDS model of simian immunodeficiency virus (SIV) infection. Here we show an increase in the *in vivo* virulence of an MHC-I-adapted virus despite a reduction in *in vitro* viral replication capacity. Only a few of the selected escape mutations reverted after transmission to MHC-I-disparate recipients. Results clearly showed that MHC-I-adapted SIVs that have been serially-transmitted through MHC-I-mismatched hosts can have higher *in vivo* virulence in MHC-I-matched hosts despite their lower *in vitro* viral fitness. This study suggests that HIVs may become less sensitive to CD8^+^ T cell responses and could have increased *in vivo* virulence by adaptation to MHC-I in human populations.

## Introduction

Human immunodeficiency virus (HIV) induces persistent viremia leading to AIDS onset in humans. Virus-specific CD8^+^ T-cell responses exert strong suppressive pressure on HIV replication [1–3] but fail to control viremia in most infections. Several human leukocyte antigen (HLA) or major histocompatibility complex (MHC) alleles have been shown to be associated with lower viral loads [4–6]. Virus control associated with some of these protective MHC class I (MHC-I) alleles has been attributed to Gag epitope-specific CD8^+^ T-cell responses [6–9]. For instance, CD8^+^ T-cell responses specific for the HLA-B*57-restricted Gag_240–249_ TW10 and HLA-B*27-restricted Gag_263–272_ KK10 epitopes exert strong suppressive pressure on HIV replication, leading to lower viral loads [10–14].

Potent HIV-specific CD8^+^ T cells select for MHC-I-associated mutations resulting in viral escape from CD8^+^ T-cell recognition often with reduced *in vitro* viral fitness [15–18]. Virus transmission to MHC-I-mismatched individuals could result in reversion of these mutations to recover viral fitness [6,17,19–21]. Thus, it has been speculated that HIV may evolve by selection of individual MHC-I-associated mutations and their reversion after multiple transmissions among individuals with highly-diversified MHC-I genotypes.

Recent studies have suggested that HIV evolves to have lower *in vitro* replication capacity through accumulation of MHC-I-associated mutations in human populations [17,22]. These studies in HIV-infected humans, however, have had difficulties in addressing the following issues. First, it is difficult to precisely trace serial HIV transmission. Second, it is difficult to compare *in vitro* viral fitness among highly-diversified HIV variants. Finally, it is difficult to evaluate the *in vivo* replication capacity of transmitted viruses. A macaque AIDS model of simian immunodeficiency virus (SIV) infection could be helpful to address these issues.

We have previously established a group of Burmese rhesus macaques sharing individual MHC-I haplotypes [23,24] and reported the discovery of a protective MHC-I haplotype *90-120-Ia* associated with lower setpoint viral loads after SIVmac239 infection [25]. SIV Gag_206-216_ and Gag_241-249_ epitope-specific CD8^+^ T-cell responses associated with *90-120-Ia* are likely responsible for this reduction in viral loads [26,27]. SIVmac239-infected *90-120-Ia*^+^ animals exhibiting persistent viremia consistently select Gag_206-216_, Gag_241-249_, Gag_373-380_, Vif_114-124_, Nef_9-19_, Nef_89-97_, and Nef_193-203_ epitope-specific CD8^+^ T-cell escape mutations by a year post-infection [28,29]. Two of these mutations, GagL216S leading to leucine-to-serine substitution at the 216th amino acid (aa) in Gag and GagD244E leading to aspartic acid-to-glutamic acid at the 244th, were shown to result in loss of viral fitness [26,27,30].

In the present study, we performed serial transmissions of SIV adapted to the protective MHC-I haplotype *90-120-Ia* through MHC-I-mismatched rhesus macaques. To determine how viruses with *90-120-Ia*-associated mutations can change after multiple transmissions, we first infected *90-120-Ia*^-^ macaques using a plasma sample from a *90-120-Ia*^+^ macaque at 1 year post-infection with the SIVmac239 clone, and performed further plasma transmission through *90-120-Ia*^-^ macaques. Our analysis revealed that the viruses passaged through *90-120-Ia*^-^ macaques maintained *90-120-Ia*-associated mutations and induced persistent viremia in *90-120-Ia*^-^ macaques despite their lower *in vitro* viral fitness. Notably, this passaged viral isolate showed rapid disease progression in *90-120-Ia*^+^ macaques when compared to wild-type SIV. These results suggest that passaged viruses can maintain escape mutations and are thus less sensitive to CD8^+^ T cells restricted by protective MHC-I alleles.

## Results

### MHC-I-associated mutations persist after serial SIV transmission

**Fig 1:**
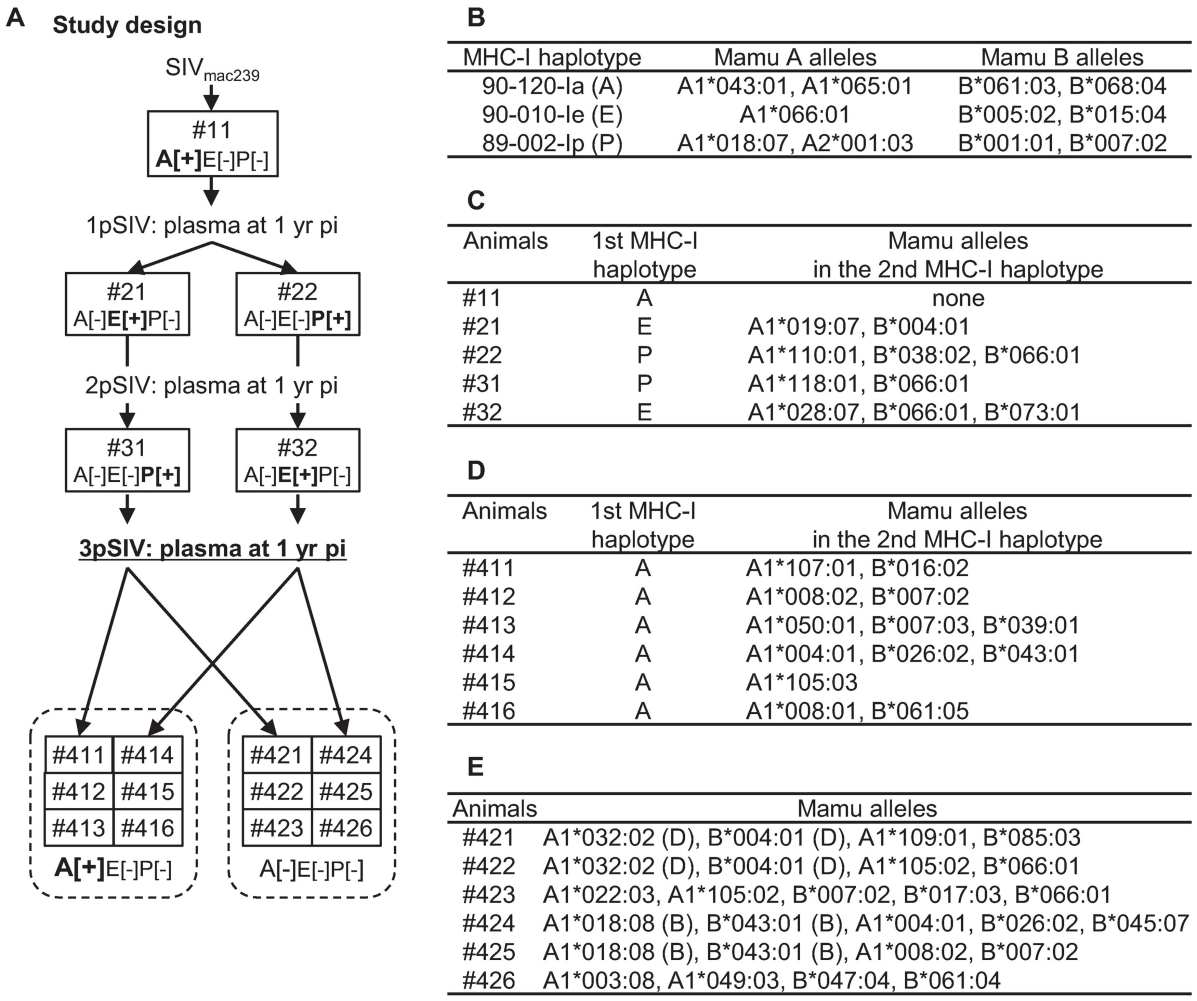
Schema for SIV transmission experiments in macaques. **A.** Study design. The first passage SIV plasma (1pSIV) was obtained from MHC-I haplotype *90-120-Ia*^+^ macaque #11 at 1 year after wild-type SIVmac239 infection. The second passage SIV plasma (2pSIV) obtained from *90-120-Ia*^-^/*90-010-Ie*^+^/*89-002-Ip*^-^ (A[–]E[+]P[–]) and *90-120-Ia*^-^/*90-010-Ie*^-^/*89-002-Ip*^+^ (A[–]E[–]P[+]) macaques (#21 and #22) at 1 year after 1pSIV infection was transmitted to A[–]E[–]P[+] and A[–]E[+]P[–] macaques (#31 and #32) to obtain the third passage SIV plasma (3pSIV) at 1 year post-infection, respectively. 3pSIV challenge experiments were performed in six A[+]E[–]P[–] and six A[–]E[–]P[–] macaques, respectively. **B.** Confirmed *Mamu-A* and *Mamu-B* alleles in MHC-I haplotypes *90-120-Ia*, *90-010-Ie*, and *89-002-Ip*. **C.** MHC-I haplotypes and confirmed *Mamu-A*/*B* alleles not included in MHC-I haplotype *90-120-Ia*, *90-010-Ie*, or *89-002-Ip* in macaques #11, #21, #22, #31, and #32 used for serial SIV transmissions. Animal #11 was indicated to have MHC-I haplotype *90-120-Ia* homozygous. **D.** Confirmed *Mamu-A/B* alleles not included in MHC-I haplotype *90-120-Ia* in A[+]E[–]P[–] macaques used for 3pSIV infection. **E.** Confirmed *Mamu-A/B* alleles in A[–]E[–]P[–] macaques used for 3pSIV infection. Macaques #421 and #422 shared the MHC-I haplotype *90-010-Id* [D] and macaques #424 and #425 shared the MHC-I haplotype *90-120-Ib* [B].

**Fig 2:**
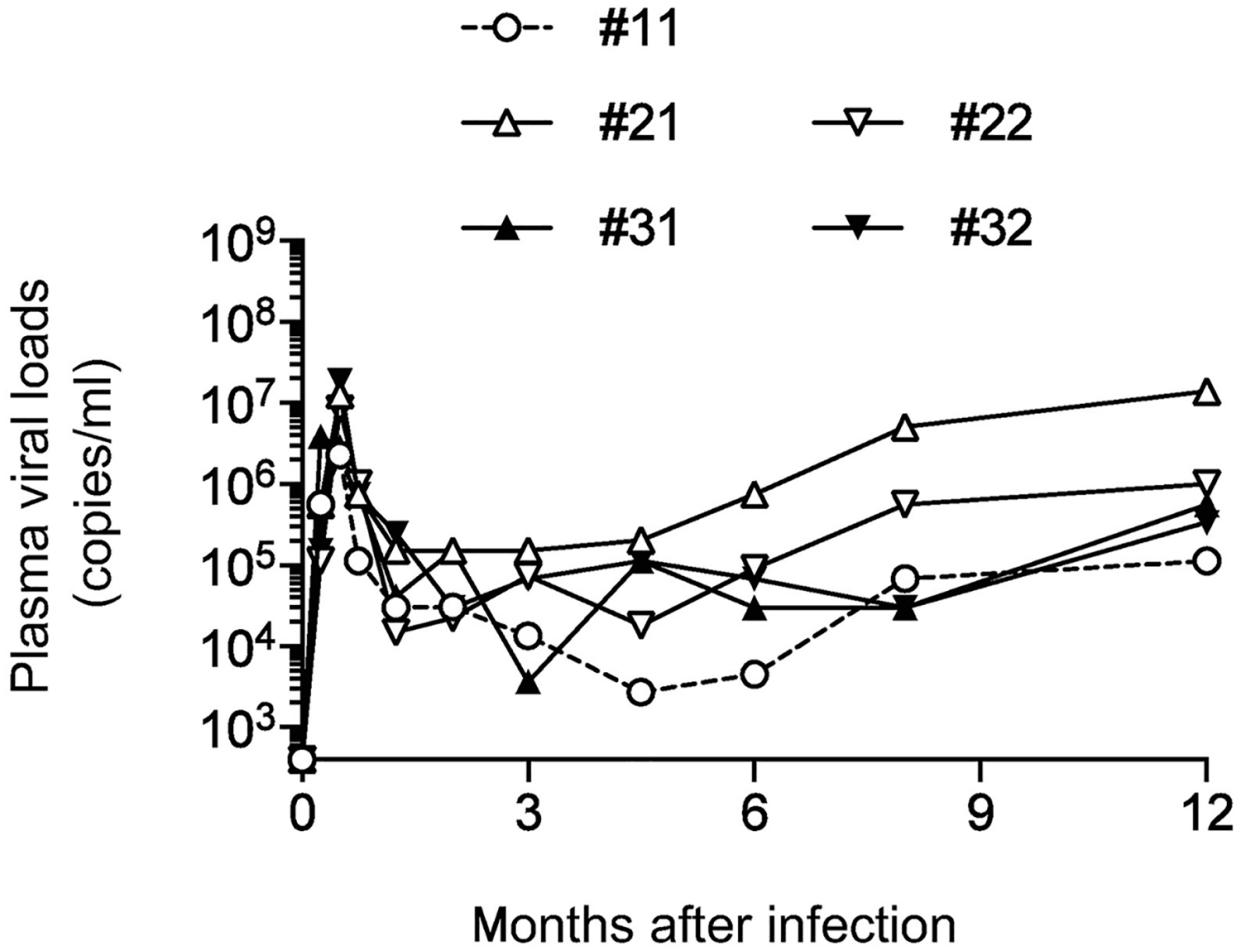
Changes in plasma viral loads in serially SIV transmitted macaques. Plasma viral loads (SIV *gag* RNA copies/ml plasma) in macaques #11, #21, #22, #31, and #32 were determined as described previously [26]. The lower limit of detection is approximately 4 x 10^2^ copies/ml. Viral loads in macaque #11 were reported previously [25].

We performed serial SIV transmissions in rhesus macaques. A plasma sample obtained from macaque #11 possessing the MHC-I haplotype *90-120-Ia* 1 year after SIVmac239 infection was used as the first passage SIV (1pSIV) (Fig 1). MHC-I *Mamu-A* and *Mamu-B* analysis detected only *90-120-Ia*-derived alleles in this animal. Macaque #11 showed persistent viremia and developed AIDS 17 months after SIVmac239 infection [25]. The 1pSIV plasma sample was inoculated intravenously (i.v.) into *90-120-Ia*^-^ macaques #21 and #22 possessing MHC-I haplotypes *90-010-Ie* and *89-002-Ip*, respectively (Fig 1). Both animals showed persistent viremia (Fig 2) and the second passage SIV plasma samples (2pSIV) were obtained 1 year after 1pSIV infection from these two macaques (2pSIV1 from macaque #21 and 2pSIV2 from #22). The 2pSIV1 and 2pSIV2 plasma samples were then inoculated i.v. into *90-120-Ia*^-^ macaques #31 and #32 possessing MHC-I haplotypes *89-002-Ip* and *90-010-Ie*, respectively (Fig 1). Both animals again showed persistent viremia (Fig 2) and the third passage SIV plasma samples (3pSIV) were obtained 1 year after 2pSIV infection from these two macaques (3pSIV1 from macaque #31 and 3pSIV2 from #32). Thus, 3pSIV1 was obtained after transmissions through #11-#21-#31 while 3pSIV2 through #11-#22-#32. No MHC-I alleles were shared among the former three animals but Mamu-B*066:*01* was shared in two (#22 and #32) of the latter three animals (Fig 1).

Viral Gag-, Pol-, Vif-, Vpx-, Vpr-, Tat-, Rev-, and Nef-coding regions in 1pSIV had dominant mutations resulting in aa substitutions at twenty-nine residues (8 in Gag, 4 in Pol, 4 in Vif, 1 in Vpx, 1 in Vpr, 5 in Rev, and 6 in Nef) as described previously [25]. These included seven mutations leading to aa substitutions at the 216th, 244th, and 375th residues in Gag, the 115th in Vif, and the 12th, 90th, and 201st in Nef, respectively. These replacements were previously shown to be selected for by CD8^+^ T cells in *90-120-Ia*^+^ macaques by 1 year after SIVmac239 infection and result in escape from recognition by Gag_206-216_, Gag_241-249_, Gag_373-380_, Vif_114-124_, Nef_9-19_, Nef_89-97_, and Nef_193-203_ epitope-specific CD8^+^ T cells, respectively [28,29].

**Fig 3:**
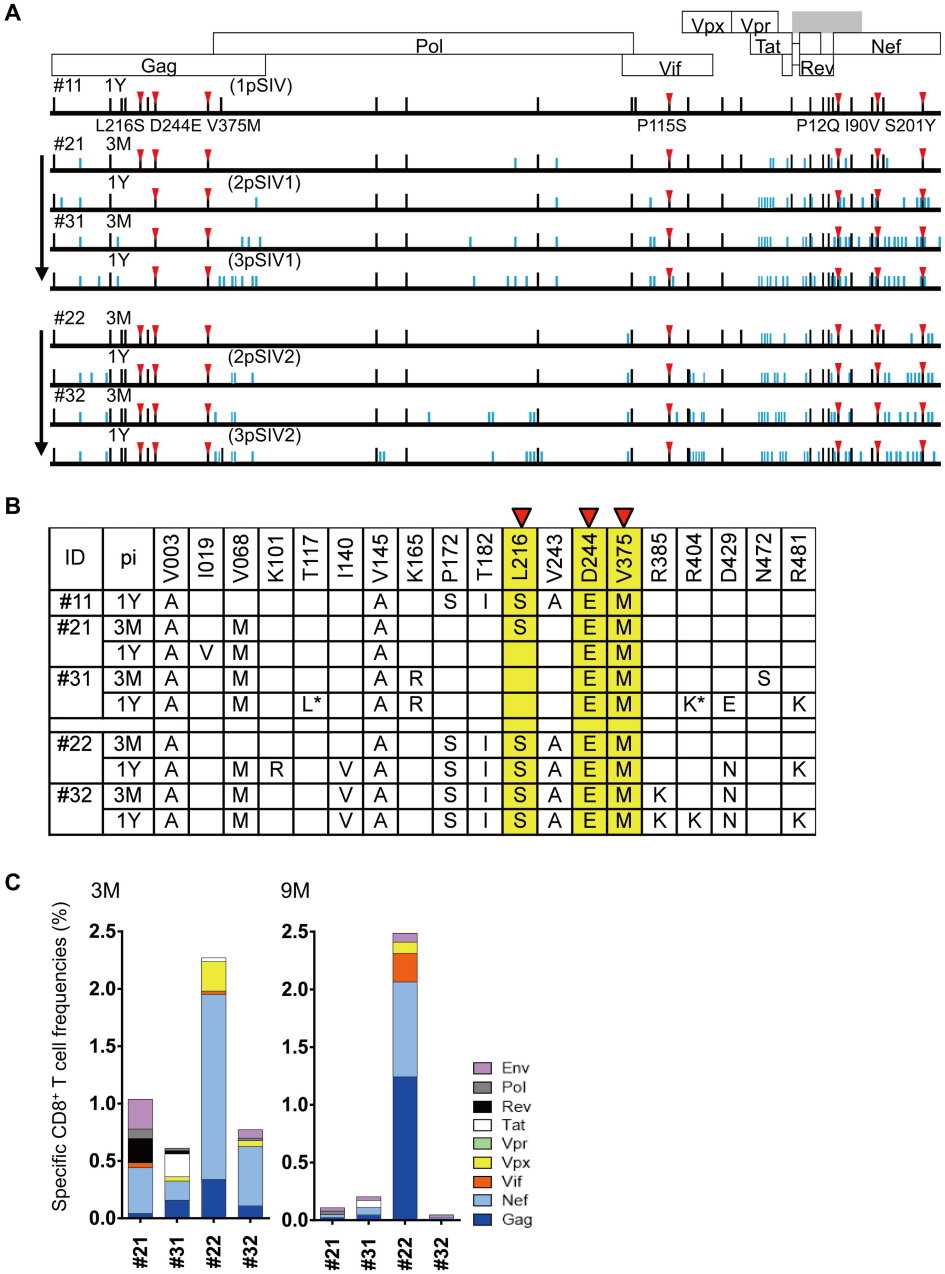
Viral genome sequences and virus-specific CD8^+^ T-cell responses in serially SIV transmitted macaques. **A.** Dominant nonsynonymous mutations in plasma viruses obtained from SIV transmitted macaques. We examined sequences of viral cDNAs encoding Gag, Pol, Vif, Vpx, Vpr, Tat, Rev, and Nef amplified from plasma RNAs obtained from macaques #21, #22, #31, and #32 at 3 months (3M) and 1 year (1Y) post-infection. The data on #11-derived plasma was previously obtained [25]. Amino acid substitutions from wild-type SIVmac239 sequences are shown by bars. Black bars indicate the mutations selected in 1pSIV (#11). Red triangles indicate the mutations resulting in escape from *90-120-Ia*-associated CD8^+^ T-cell recognition. **B.** Dominant nonsynonymous *gag* mutations in plasma viruses obtained from SIV transmitted macaques. Among the mutations described in the above Fig 3A, amino acid substitutions in Gag are shown. **C.** SIV antigen-specific CD8^+^ T-cell responses in macaques #21, #22, #31, and #32 at 3 and 9 months post-infection.

Of the twenty-nine mutations selected in 1pSIV, nine reverted in macaque #21 followed by one additional reversion and one re-selection in #31. Six reverted in macaque #22 but two were selected again in #32. Thus, twenty and twenty-five of twenty-nine mutations selected in 1pSIV remained in 3pSIV1 and 3pSIV2, respectively (Fig 3A and 3B and S1 Fig). Regarding the seven *90-120-Ia*-associated CD8^+^ T-cell escape mutations described above, six remained without reversion even in 3pSIV1 and 3pSIV2. The GagL216S mutation reverted in macaque #21 but was maintained in macaques #22 and #32, while both 3pSIV1 and 3pSIV2 still had the GagD244E. Thus, the majority of *90-120-Ia*-associated mutations remained without reversion even in 3pSIV through two passages. Macaques #21, #22, #31, and #32 elicited CD8^+^ T-cell responses targeting multiple SIV antigens (Fig 3C). All of these four animals exhibited high frequency Nef-specific CD8^+^ T-cell responses. Several mutations in addition to the twenty-nine selected in 1pSIV were selected in macaques #21-#31 and #22-#32 (Fig 3A).

Next generation sequencing (NGS) confirmed viral diversification in our transmitted plasma samples (S2 and S3 Figs). Phylogenetic distances of viral Gag CA-coding region from wild-type SIVmac239 decreased in macaque #21 but increased in macaques #31, #22, and #32, which may reflect the limited pressure exerted by Gag-specific CD8^+^ T-cell responses in #21 (Fig 3C). Phylogenetic distances of Vif-coding and Nef-coding regions from wild-type SIVmac239 increased in individual animals. Changes in viral genome sequences were the largest in the Nef-coding region, possibly reflecting larger CD8^+^ T-cell responses targeting Nef (Fig 3C).

### 3pSIV exhibits lower *in vitro* viral fitness

We then attempted to compare the *in vitro* replication capacity of wild-type SIVmac239 and the passaged viruses 1pSIV, 2pSIVs, and 3pSIVs. It is not easy to compare *in vitro* replication capacity of plasma HIVs directly and previous studies mostly used recombinant viruses derived from molecular clones such as NL4-3 where *gag* is replaced by the predominant plasma HIV sequences for comparison of *in vitro* viral fitness [17,22]. In the present study, we examined the *in vitro* replication capacity of viruses recovered from peripheral blood mononuclear cells (PBMCs) and plasma as well as recombinant SIVmac239-derived viruses whose *gag* was replaced by the predominant plasma SIV sequences.

**Fig 4:**
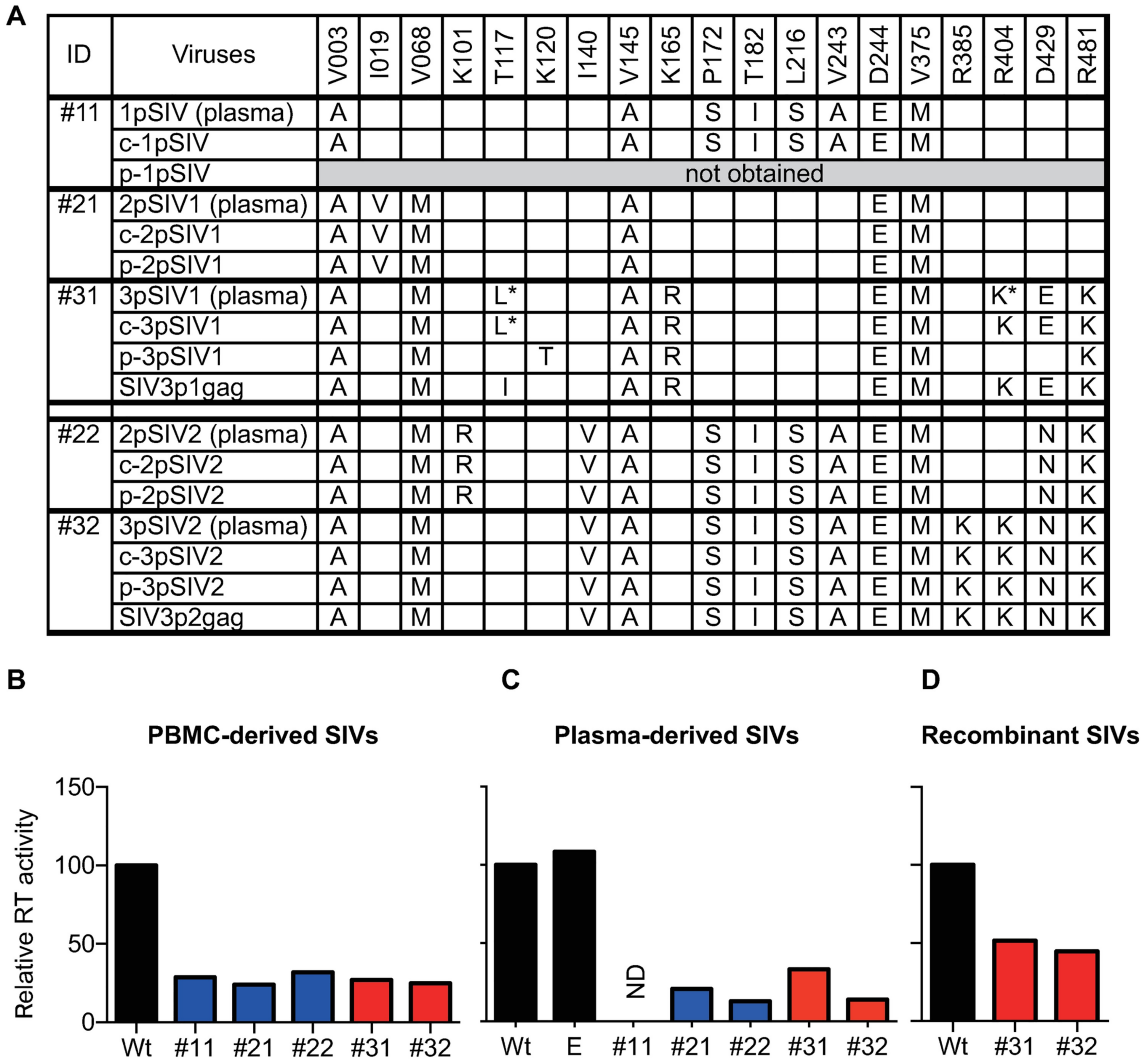
Comparison of *in vitro* replication capacity of passaged viruses. **A.** Dominant nonsynonymous *gag* mutations in recovered viruses. We recovered viruses, c-1pSIV, c-2pSIV1, c-2pSIV2, c-3pSIV1, and c-3pSIV2, from PBMCs obtained at 1 year post-infection from macaques #11, #21, #22, #31, and #32, respectively. We also recovered viruses, p-2pSIV1, p-2pSIV2, p-3pSIV1, and p-3pSIV2, from 2pSIVs or 3pSIVs plasma but failed to recover viruses from 1pSIV plasma. In addition, we constructed recombinant SIVs, SIV3p1gag and SIV3p2gag, carrying 3pSIV1-derived and 3pSIV2-derived *gag*, respectively. **B. C. D.** Relative RT activity of the HSC-F cell culture supernatants on day 4 after infection with PBMC-derived **(B)**, plasma-derived **(C)**, and recombinant SIVs **(D)**. The "E" in the graph **C** indicates the data on infection with the virus derived from plasma obtained at 1 year after SIVmac239 infection from a *90-120-Ia*^-^ but *90-010-Ie*^+^ macaque (R08-014) that was used in our previous experiment [42]. Similar RT levels were detected in the supernatants of cells infected with the wild-type SIV and the E plasma-derived virus. The E plasma-derived virus had two dominant nonsynonymous mutations in *gag* at the positions encoding the residues 129 (the mutation/wild-type ratio: more than 4/1) and 468 (more than 1/1 but less than 4/1), but these mutations did not result in loss of *in vitro* viral fitness. RT activities relative to that of the wild type (set at 100) are shown. Representative results from two sets of experiments are shown.

First, PBMCs from macaques #11, #21, #22, #31, and #32 at 1 year post-infection were cultured to obtain PBMC-derived virus stocks, referred to as c-1pSIV, c-2pSIV1, c-2pSIV2, c-3pSIV1, and c-3pSIV2, respectively. These viruses had the same nonsynonymous *gag* mutations with those in *gag* cDNAs amplified from plasma RNAs at 1 year post-infection (Fig 4A). The culture supernatants of HSC-F cells (a macaque T cell line) on day 4 after infection with these PBMC-derived viruses showed lower reverse transcription (RT) activity compared to the wild-type SIVmac239 (Fig 4B). These results indicate that all the PBMC-derived viruses, c-1pSIV, c-2pSIV1, c-2pSIV2, c-3pSIV1, and c-3pSIV2, have lower *in vitro* replicative capacities compared to wild-type SIV.

Second, HSC-F cells infected with concentrated plasma samples obtained from macaques #21, #22, #31, and #32 at 1 year post-infection were cultured to obtain the culture supernatants as passaged plasma-derived virus stocks, referred to as p-2pSIV1, p-2pSIV2, p-3pSIV1, and p-3pSIV2, respectively. We failed to recover a plasma-derived virus stock from macaque #11. There were a few differences between p-3pSIV1-derived and plasma RNA-derived *gag* sequences, but p-2pSIV1, p-2pSIV2, and p-3pSIV2 had the same nonsynonymous *gag* mutations to those in *gag* cDNAs amplified from plasma RNAs at 1 year post-infection (Fig 4A). Again, all of these virus-infected HSC-F cultures showed lower RT activity compared to the wild-type SIVmac239 in the culture supernatants on day 4 after infection (Fig 4C).

Furthermore, we compared *in vitro* viral fitness of these viruses with the wild-type SIV by competition assay. For comparison of wild-type and passaged viruses, HSC-F cells infected with individual virus stocks were cocultured to determine which viral genome sequences become dominant in the culture supernatants. In competition assay of wild-type SIVmac239 with any of the PBMC-derived virus stocks, the wild-type sequences became dominant (S4 Fig). Competition assay using plasma-derived virus stocks confirmed the results obtained from the PBMC-derived virus stocks (S4 Fig). These results indicate lower *in vitro* replication capacity of passaged viruses compared to the wild-type SIV.

Finally, we constructed SIVmac239-derived recombinant viruses, SIV3p1gag and SIV3p2gag, where *gag* was replaced by the predominant 3pSIV1 and 3pSIV2 sequences, respectively (Fig 4A). RT assay of the culture supernatants of HSC-F cells on day 4 after infection revealed lower *in vitro* viral fitness of both of these recombinant viruses compared to the wild-type SIVmac239 (Fig 4D).

### 3pSIV induces more rapid disease progression in *90-120-Ia*^+^ macaques

Next, we used the 3pSIV1/2 plasma samples to challenge six *90-120-Ia*^+^/*90-010-Ie*^-^/*89-002-Ip*^-^ (A[+]E[–]P[–]) and six *90-120-Ia*^-^/*90-010-Ie*^-^/*89-002-Ip*^-^ (A[–]E[–]P[–]) macaques. Three A[+]E[–]P[–] macaques #411, #412, and #413 and three A[–]E[–]P[–] macaques #421, #422, and #423 were intravenously inoculated with 3pSIV1, whereas three A[+]E[–]P[–] macaques #414, #415, and #416 and three A[–]E[–]P[–] macaques #424, #425, and #426 were intravenously inoculated with 3pSIV2 (Fig 1).

**Fig 5:**
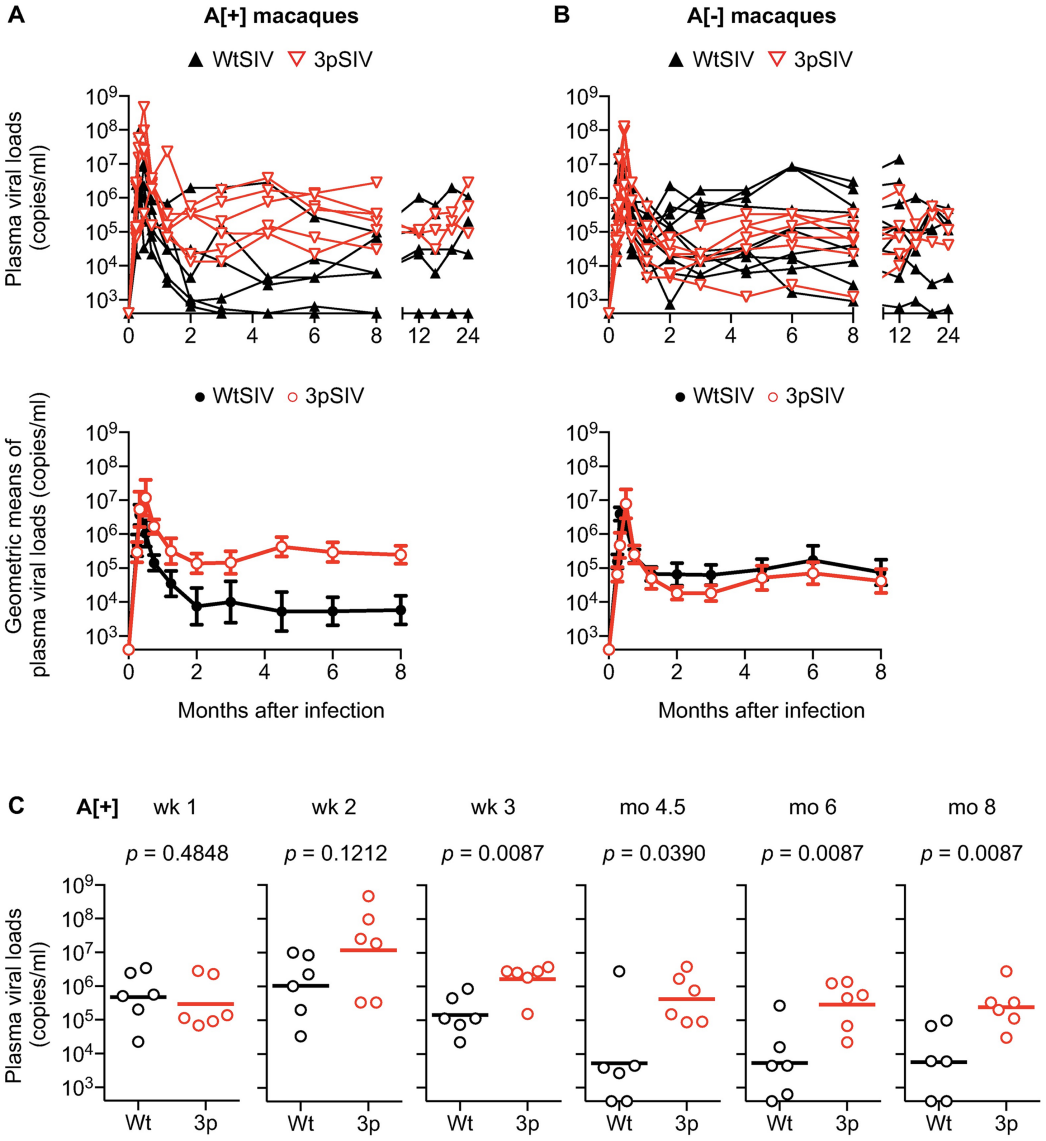
Comparison of plasma viral loads after wild-type SIV (WtSIV) and 3pSIV infection. **A.** Changes in viral loads in *90-120-Ia*^+^ macaques infected with WtSIV (*n* = 6, black) or 3pSIV (*n* = 6, red). Plasma viral loads (SIV *gag* RNA copies/ml plasma) were determined as described previously [26]. The lower limit of detection is approximately 4 x 10^2^ copies/ml. The upper panel shows data on individual animals and the lower geometric means. **B.** Changes in viral loads in *90-120-Ia*^-^ macaques infected with WtSIV (*n* = 10, black) or 3pSIV (*n* = 6, red). The upper panel shows data on individual animals and the lower geometric means. No significant difference was observed in *90-120-Ia*^-^ macaques between WtSIV and 3pSIV infection. **C.** Comparison of viral loads in *90-120-Ia*^+^ macaques in the acute phase (at weeks [wk] 1, 2, and 3) and at the setpoint (at months [mo] 4.5, 6, and 8) after WtSIV and 3pSIV infection. 3pSIV-infected animals showed significantly higher viral loads than WtSIV not at wk 1 or wk 2 but at wk 3, mo 4.5, mo 6, and mo 8 (by Mann-Whitney U-test). Data on viral loads in WtSIV-infected *90-120-Ia*^+^ and *90-120-Ia*^-^ macaques were previously reported [25].

All the 3pSIV-infected animals showed persistent viremia (Fig 5A and 5B), despite the lower *in vitro* viral fitness of 3pSIV1/3pSIV2. No clear difference was observed in viral loads between 3pSIV1 and 3pSIV2 infection in either A[+]E[–]P[–] or A[–]E[–]P[–] macaques. Furthermore, no significant difference was observed in viral loads between 3pSIV-infected *90-120-Ia*^-^ macaques and the SIVmac239-infected *90-120-Ia*^-^ control group (n = 10) consisting of *90-010-Ie*^+^ (n = 6) and *90-120-Ib*^+^ (n = 4) animals that were previously reported to show typical levels of viremia [25]. Information on Mamu-A/B alleles in the control group was described in the previous report [25].

In contrast, 3pSIV-infected *90-120-Ia*^+^ macaques exhibited significantly higher viral loads at week 3 post-infection compared with those in previously-reported wild-type SIVmac239-infected *90-120-Ia*^+^ macaques (n = 6) [25] (p = 0.0087 by Mann-Whitney U-test) (Fig 5C). Interestingly, no significant difference in viral loads at week 1 was observed between these two groups (Fig 5C and S5 Fig). Remarkably, 3pSIV-infected *90-120-Ia*^+^ macaques showed significantly higher viral loads at months 4.5, 6, and 8 post-infection than the SIVmac239-infected *90-120-Ia*^+^ macaques (p = 0.0390 at month 4.5, p = 0.0087 at month 6, and p = 0.0087 at month 8) (Fig 5C). This suggests that 3pSIV infection results in significantly higher setpoint viral loads in *90-120-Ia*^+^ macaques than wild-type SIVmac239 does.

**Fig 6:**
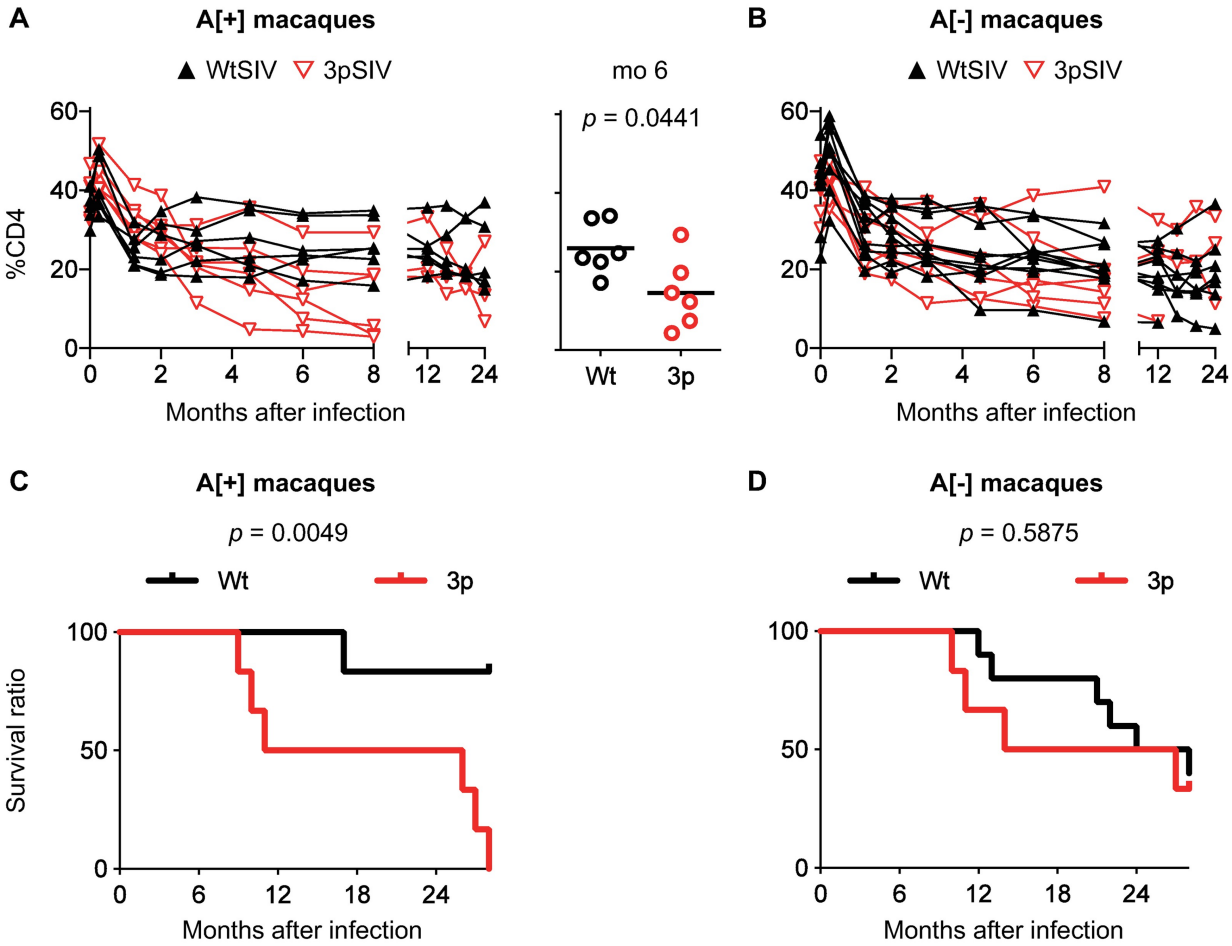
Comparison of %CD4 and survival periods after WtSIV and 3pSIV infection. **A.** Changes in peripheral %CD4^+^ T cells in *90-120-Ia*^+^ macaques infected with WtSIV (*n* = 6, black) or 3pSIV (*n* = 6, red). Comparison at 6 months post-infection showed significantly lower %CD4 in 3pSIV-infected macaques than in WtSIV (by Mann-Whitney U-test). **B.** Changes in peripheral %CD4^+^ T cells in *90-120-Ia*^-^ macaques infected with WtSIV (*n* = 10, black) or 3pSIV (*n* = 6, red). No significant difference was observed in *90-120-Ia*^-^ macaques between WtSIV and 3pSIV infection. **C.** Kaplan-Meier survival curves in *90-120-Ia*^+^ macaques after infection with WtSIV (*n* = 6, black) or 3pSIV (*n* = 6, red). Comparison of two curves indicated significantly shorter survival periods in 3pSIV-infected animals than WtSIV-infected (chi square, 7.898; *p* = 0.0049 by log-rank test). **D.** Kaplan-Meier survival curves in *90-120-Ia*^-^ macaques after infection with WtSIV (*n* = 10, black) or 3pSIV (*n* = 6, red). Comparison of two curves indicated no significant difference between WtSIV-infected and 3pSIV-infected animals. Data in WtSIV-infected *90-120-Ia*^+^ and *90-120-Ia*^-^ macaques were previously obtained [25].

3pSIV infection showed significantly lower %CD4 at month 6 (p = 0.0441 by Mann-Whitney U-test) and shorter survival periods (p = 0.0049 by Log-rank test) than SIVmac239 in *90-120-Ia*^+^ macaques (Fig 6). Indeed, three of six 3pSIV-infected *90-120-Ia*^+^ macaques but none of the six SIVmac239-infected developed AIDS and had to be euthanized by a year post-infection, demonstrating that 3pSIV is more virulent than the wild-type SIVmac239 in *90-120-Ia*^+^ macaques. One of the *90-120-Ia*^+^ macaques, #412, shared a *89-002-Ip*-derived MHC-I allele Mamu-B*007:*02* with macaque #31. However, the five other 3pSIV-infected *90-120-Ia*^+^ macaques (excluding #412) still showed significantly higher setpoint viral loads and shorter survival periods than SIVmac239-infected *90-120-Ia*^+^ macaques (S6 Fig). In *90-120-Ia*^-^ macaques, no significant difference was observed in %CD4 nor survival periods between 3pSIV and SIVmac239 (Fig 6), although the former showed lower *in vitro* viral fitness when compared to the latter.

**Fig 7:**
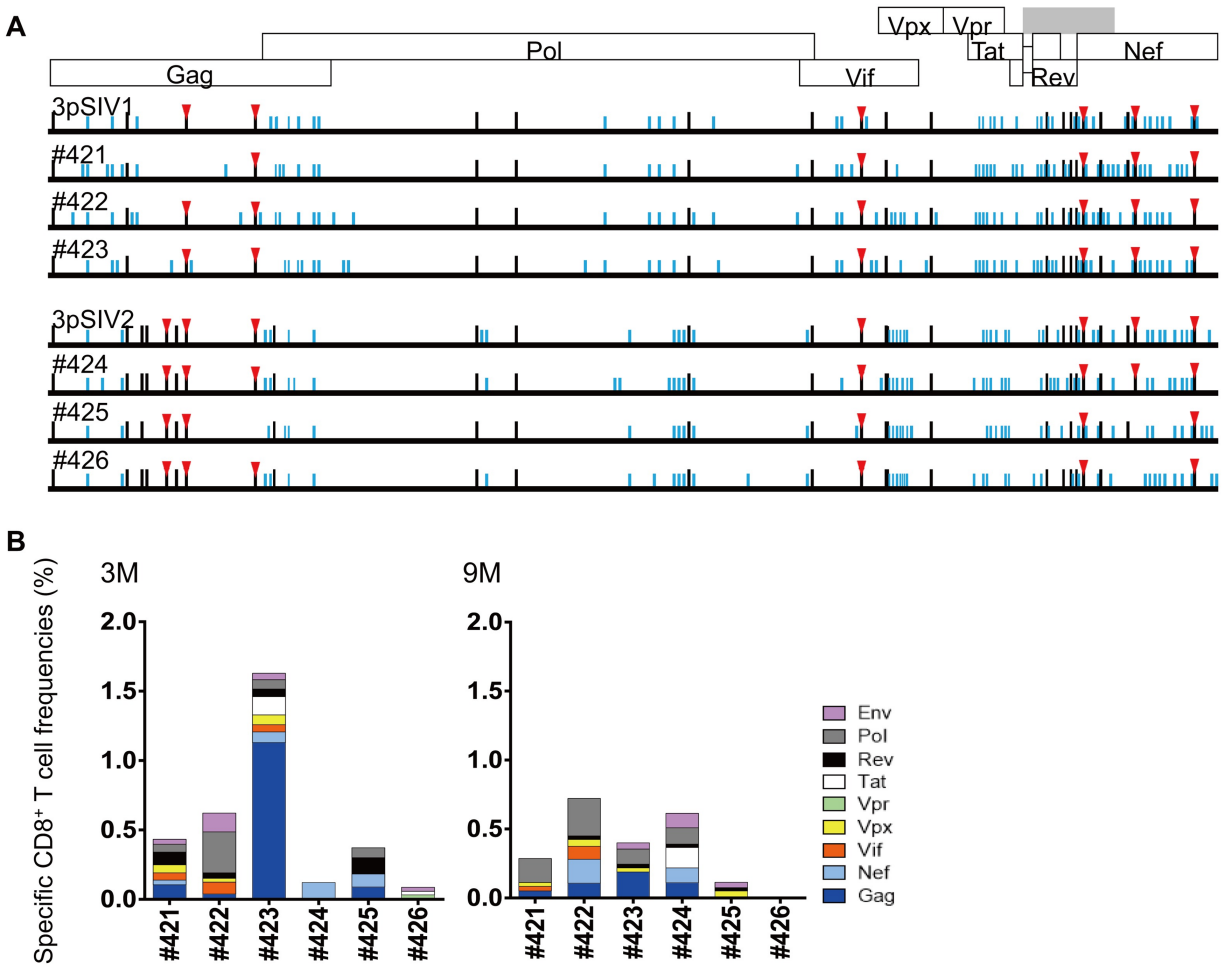
Viral genome sequences and virus-specific CD8^+^ T-cell responses in 3pSIV-infected *90-120-Ia*^-^ macaques. **A.** Dominant nonsynonymous mutations in plasma viruses obtained from 3pSIV-infected *90-120-Ia*^-^ macaques at 1 year (in #421, #422, #423, and #424) or at euthanasia 10–11 months (in #425 and #426) post-infection. Amino acid substitutions from wild-type SIVmac239 sequences are shown by bars. Black bars indicate the mutations selected in 1pSIV. Red triangles indicate the mutations resulting in escape from *90-120-Ia*-associated CD8^+^ T-cell recognition. **B.** SIV antigen-specific CD8^+^ T-cell responses in 3pSIV-infected *90-120-Ia*^-^ macaques at 3 and 9 months after 3pSIV infection.

The *90-120-Ia*^-^ macaques infected with 3pSIV with twenty or twenty-five of the twenty-nine mutations selected in 1pSIV showed one to five reversions and maintained no less than nineteen of them at 1 year post-infection (Fig 7). Regarding the seven *90-120-Ia*-associated CD8^+^ T-cell escape mutations, no reversion was observed in three (#422, #423, and #424) of the six animals, one in two (#421 and #426), and two in one (#425).

**Fig 8:**
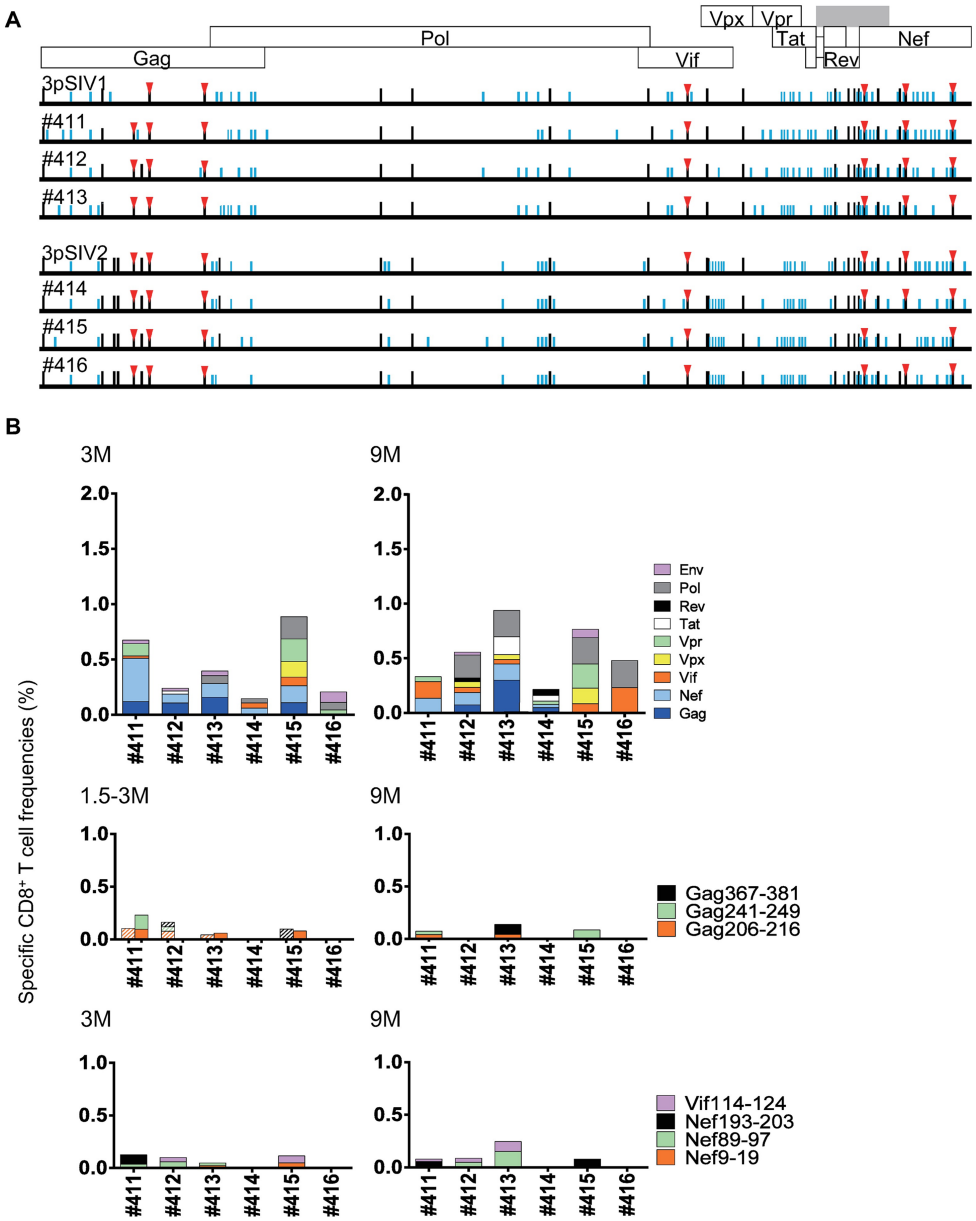
Viral genome sequences and virus-specific CD8^+^ T-cell responses in 3pSIV-infected *90-120-Ia*^+^ macaques. **A.** Dominant nonsynonymous mutations in plasma viruses obtained from 3pSIV-infected *90-120-Ia*^+^ macaques at 1 year (in #411, #414, and #415) or at euthanasia 9–11 months (in #412, #413, and #416) post-infection. Amino acid substitutions from wild-type SIVmac239 sequences are shown by bars. Black bars indicate the mutations selected in 1pSIV. Red triangles indicate the mutations resulting in escape from *90-120-Ia*-associated CD8^+^ T-cell recognition. **B.** CD8^+^ T-cell responses specific for SIV antigens (top panels) or *90-120-Ia*-associated CD8^+^ T-cell epitope peptides (middle and bottom panels) in 3pSIV-infected *90-120-Ia*^+^ macaques at 3 and 9 months after 3pSIV infection. In the middle panel, data at 1.5 (striped) and 3 months (shaded) post-infection are shown.

On the other hand, the *90-120-Ia*^+^ macaques infected with 3pSIV had no less than twenty-one of the twenty-nine mutations selected in 1pSIV at 1 year post-infection (Fig 8A). Regarding the seven *90-120-Ia*-associated CD8^+^ T-cell escape mutations, GagL216S was again selected for in all the three of the 3pSIV1-infected animals, and all seven mutations were dominant in the six 3pSIV-infected *90-120-Ia*^+^ macaques except for #415 which showed a reversion at the 90th residue in Nef (Fig 8A). Indeed, Gag_206-216_, Gag_241-249_, Gag_373-380_, Vif_114-124_, Nef_9-19_, Nef_89-97_, and Nef_193-203_ epitope-specific CD8^+^ T-cell responses were low frequency in these *90-120-Ia*^+^ animals (Fig 8B). Results suggest that these escape mutations in 3pSIV were maintained in *90-120-Ia*^+^ macaques.

## Discussion

HIV induces persistent infection and accumulates viral mutations largely due to selection by CD8^+^ T cells. These mutations often have viral fitness costs and some of them can revert after viral transmission into MHC-I-mismatched hosts [6,17,19–21]. Recent studies in HIV-infected individuals have suggested that these MHC-I-associated mutations can accumulate in the population [22,31,32]. Analysis of HIV-infected transmission pairs has indicated that transmission of HIV mutations associated with the recipients' MHC-I alleles can result in higher viral loads [33,34]. Our present study in a macaque AIDS model demonstrated direct evidence indicating that MHC-I-adapted viruses that have been serially-passaged through MHC-I-mismatched hosts, even with lower *in vitro* viral fitness, can induce higher viral loads and more rapid disease progression in MHC-I-matched hosts.

The majority of the mutations selected for in *90-120-Ia*^+^ animal #11 were present in the 3pSIV virus isolate. Analysis of viral genome sequences in 3pSIV-infected *90-120-Ia*^-^ animals showed that the majority of the protective MHC-I haplotype *90-120-Ia*-associated escape mutations were maintained after three serial passages. These mutations were preserved after three transmissions in *90-120-Ia*^-^ animals, supporting the notion that MHC-I-associated mutations can be maintained in circulating viruses in populations. The *90-120-Ia*-associated mutations include GagL216S and GagD244E resulting in reduction of *in vitro* viral fitness [26,27]. Rapid reversion of these mutations was consistently observed after infection with SIV containing a single mutation in our previous study [35]. However, reversion occurred only rarely in infection with SIV carrying multiple mutations in the present study. No evidence of compensatory mutations which might have rescued viral fitness was found. After transmission, it may be more difficult for a virus with multiple prior CD8^+^ T-cell escape mutations to revert to wild-type when compared to a virus with a single escape mutation. The new host’s CD8^+^ T-cell response may exert the most important selective pressure on the multiply previously escaped virus (selected for by the prior host’s CD8^+^ T cells) and selection for the new host’s MHC-I-associated escape variants may occur first. These new CD8^+^ T-cell escape variants may have a greater selective advantage *in vivo* than any reversion of the previous host’s MHC-I-associated mutations and thus these changes will occur first perhaps delaying the reversion of the prior escape mutations.

Previous studies examined the effect of viral genome mutations on *in vitro* viral fitness in the context of molecular HIV clones such as NL4-3 [17,22]. In the present study, we constructed recombinant viruses carrying 3pSIV-derived *gag* in the context of wild-type SIVmac239 and showed that these new recombinant viruses had lower *in vitro* replicative capacities when compared to the wild-type SIVmac239. Mutations in *gag* appeared to have greater suppressive impact on *in vitro* viral fitness than those in other regions, consistent with previous reports [15–18]. Furthermore, we confirmed lower *in vitro* replication capacity of viruses recovered from plasma and PBMCs. Our results indicate that viruses carrying multiple MHC-I-associated mutations with lower *in vitro* viral fitness can be serially transmitted through MHC-I-mismatched hosts with maintaining the potential for higher viral loads in MHC-I-matched hosts. This suggests that MHC-I-adapted viruses can circulate in the population.

3pSIV obtained by serial passage through *90-120-Ia*^-^ macaques maintained several of the mutations selected for in the *90-120-Ia*^+^ macaque #11 and induced higher viral loads and more rapid disease progression in *90-120-Ia*^+^ hosts. These results demonstrate that MHC-I-adapted viruses can maintain the potential for higher virulence in MHC-I-matched hosts after serial transmissions through MHC-I-mismatched hosts. Although HIV transmissions from individuals with protective MHC-I alleles may be less efficient compared to those without protective MHC-I alleles, this study suggests that HIV isolates that are less sensitive to protective MHC-I alleles can be maintained and circulate in human populations.

3pSIV-infected *90-120-Ia*^+^ macaques appeared to generate fewer mutations than wild-type SIVmac239 post-infection, and it is speculated that there were only a limited number of CD8^+^ T-cell targets in 3pSIV-infected *90-120-Ia*^+^ macaques. This may be analogous to the situation in HIV-infected individuals where MHC-I homozygotes exhibit a more rapid course of disease progression [36]. Indeed, all of the 3pSIV-infected *90-120-Ia*^+^ animals developed AIDS in 28 months post-infection, whereas 30–40% of *90-120-Ia*^-^ animals were alive without AIDS onset at 28 months after wild-type SIVmac239 or 3pSIV infection.

In summary, we directly showed the impact of viral adaptation to MHC-I alleles on viral replication capacity *in vivo*. Protective MHC-I-adapted SIVs serially-passaged through MHC-I-mismatched hosts exhibited higher virulence in MHC-I-matched hosts despite their lower *in vitro* viral fitness. Our results indicate that MHC-I-adapted HIVs can circulate in populations, possibly resulting in loss of virus-sensitive MHC-I alleles in these populations.

## Materials and methods

### Ethics statement

Animal experiments were carried out in the Institute for Virus Research, Kyoto University (IVRKU) and Tsukuba Primate Research Center, National Institutes of Biomedical Innovation, Health and Nutrition (NIBIOHN) with the help of the Corporation for Production and Research of Laboratory Primates after approval by the Committee on the Ethics of Animal Experiments of IVRKU and NIBIOHN (permission number: R13-11, DS21-27, DS23-19, DS26-20, and DS28-18) under the guidelines for animal experiments at IVRKU, NIBIOHN, and National Institute of Infectious Diseases in accordance with the Guidelines for Proper Conduct of Animal Experiments established by Science Council of Japan (http://www.scj.go.jp/ja/info/kohyo/pdf/kohyo-20-k16-2e.pdf). The experiments were in accordance with the "Weatherall report for the use of non-human primates in research" recommendations (https://royalsociety.org/topics-policy/publications/2006/weatherall-report/). Animals were housed in adjoining individual primate cages allowing them to make sight and sound contact with one another for social interactions, where the temperature was kept at 25°C with light for 12 hours per day. Animals were fed with apples and commercial monkey diet (Type CMK-2, Clea Japan, Inc.). Blood collection and virus inoculation were performed under ketamine anesthesia. Animals were euthanized at the end of experiments or at the endpoint determined by typical signs of AIDS including reduction in peripheral CD4^+^ T-cell counts (less than 200 cells/μl), 10% loss of body weight, diarrhea, and general weakness. At euthanasia, animals were deeply anesthetized with pentobarbital under ketamine anesthesia, and then, whole blood was collected from left ventricle.

### Animal experiments

We performed serial SIV transmissions in Burmese rhesus macaque (*Macaca mulatta*). A plasma obtained from macaque #11 possessing MHC-I haplotype *90-120-Ia* at 1 year after SIVmac239 infection in our previous study [25] was used as the first passage SIV (1pSIV) (Fig 1). In the present study, 0.2 ml of 1pSIV plasma was intravenously inoculated into *90-120-Ia*^-^ macaques #21 and #22 possessing MHC-I haplotypes *90-010-Ie* and *89-002-Ip*, respectively, and the second passage SIV plasma (2pSIV) was obtained at 1 year after 1pSIV infection from these two macaques (2pSIV1 from macaque #21 and 2pSIV2 from #22). Then, 0.2 ml of 2pSIV1 and 2pSIV2 plasma were intravenously inoculated into *90-120-Ia*^-^ macaques #31 and #32 possessing MHC-I haplotypes *89-002-Ip* and *90-010-Ie*, respectively, and the third passage SIV plasma (3pSIV) was obtained at 1 year after 2pSIV infection from these two macaques (3pSIV1 from macaque #31 and 3pSIV2 from #32).

To investigate *in vivo* replication capacity of 3pSIV, 0.2 ml of 3pSIV1 was intravenously inoculated into three *90-120-Ia*^+^/*90-010-Ie*^-^/*89-002-Ip*^-^ (A[+]E[–]P[–]) macaques #411, #412, and #413 and three *90-120-Ia*^-^/*90-010-Ie*^-^/*89-002-Ip*^-^ (A[–]E[–]P[–]) macaques #421, #422, and #423, while 0.2 ml of 3pSIV2 was intravenously inoculated into three A[+]E[–]P[–] macaques #414, #415, and #416 and three A[–]E[–]P[–] macaques #424, #425, and #426 (Fig 1). The data on six *90-120-Ia*^+^ and ten *90-120-Ia*^-^ macaques intravenously infected with wild-type SIVmac239 (Figs 5 and 6; S5 and S6 Figs) were obtained in our previous study [25].

The determination of macaque MHC-I haplotypes was based on the family study in combination with the reference strand-mediated conformation analysis of *Mamu-A* and *Mamu-B* genes and detection of major *Mamu-A* and *Mamu-B* alleles by cloning the RT-PCR products as described before [24]. Confirmed MHC-I alleles consisting of MHC-I haplotypes *90-120-Ia*, *90-010-Ie*, and *89-002-Ip* were described before [24,25].

### Analysis of viral genome sequences

Viral RNAs were extracted from plasma using the High Pure Viral RNA kit (Roche). Fragments of cDNAs encoding SIVmac239 (GenBank accession number M33262) Gag, Pol, Vif, Vpx, Vpr, Tat, Rev, and Nef were amplified from plasma RNAs by nested RT-PCR and subjected to direct sequencing by using dye terminator chemistry and an automated DNA sequencer (Applied Biosystems) as described before [28]. Predominant nonsynonymous mutations were determined. The Env-coding region known to have multiple antibody-related mutations was not included in the analysis.

For pyrosequencing, cDNA fragments corresponding to nucleotides (nt) 1760–2463 (containing entire Gag capsid [CA]-coding region), nt 5460–6340 (containing entire Vif-coding region), and nt 9257–10167 (containing entire Nef-coding region) were used for making fragmentation libraries using GS FLX Titanium Rapid Library Preparation Kit (Roche). The products were cleaned with Agencourt AMPure XP magnetic beads (Beckman Coulter) followed by quality control using Agilent 2100 Bioanalyzer (Agilent Technologies). Emulsion PCR was performed with GS junior Titanium emPCR Kit Lib-L (Roche). The emPCR products were deposited onto a GS Junior Titanium Pico Titer Plate and sequenced on the GS Junior System (Roche). Sequencing reads were analyzed by the GS Amplicon Variant Analyzer Software (Roche). After alignment of the FASTA files, populations of <1% were excluded. Molecular phylogenetic analyses were conducted by the Maximum Likelihood method using the MEGA6 software (http://www.megasoftware.net/).

### Analysis of *in vitro* viral fitness

We recovered virus stocks from PBMCs and concentrated plasma samples obtained from macaques #11, #21, #22, #31, and #32 at 1 year post-infection. First, 1–5 x10^5^ CD8^-^ T cells negatively-selected from PBMCs were cultured in RPMI with 10% fetal bovine serum and 10 ng/ml human interleukin-2 (hIL-2) (Roche) with stimulation by 2 μg/ml Phytohemagglutinin-L (Sigma) on the first 2 days. The culture supernatants on day 6 were added into HSC-F cells (a cynomolgus macaque T-cell line) [37], which were cultured for 5–7 days to obtain the culture supernatants as PBMC-derived virus stocks. Second, plasma samples were concentrated by 6-fold using Lenti-X Concentrator (Clontech) and cocultured with HSC-F cells for 6–14 days to obtain the culture supernatants as plasma-derived virus stocks. To prepare the wild-type virus stock, we first obtained culture supernatants from MT4 cells (a human T-cell line) expressing CCR5 after transfection with the wild-type SIVmac239 molecular clone DNA (pBRmac239) [38]. Then, these supernatants were added into HSC-F cells and the culture supernatants were obtained as the wild-type SIVmac239 stock, which was used for comparison of *in vitro* viral fitness with PBMC-derived and plasma-derived viruses. In addition, we constructed recombinant SIV clones by replacing the *gag* region (nt 1056–3408) in the wild-type pBRmac239 molecular clone with that amplified from plasma RNAs of macaques #31 and #32 at 1 year post-infection. We then obtained recombinant molecular clones whose *gag* had the predominant 3pSIV1 and 3pSIV2 sequences, respectively. COS-1 cells were transfected with these molecular clones to obtain recombinant SIV3p1gag and SIV3p2gag virus stocks. COS-1 cells were transfected with pBRmac239 to obtain the wild-type SIVmac239 stock used for comparison of *in vitro* viral fitness with these recombinant viruses, SIV3p1gag and SIV3p2gag. Titers of these virus stocks were measured by RT assay as described previously [39,40]. For analysis of *in vitro* replication capacity, HSC-F cells were infected with these viruses (5 x 10^4^ HSC-F cells were infected with viruses having the same RT activity with the wild-type SIVmac239 corresponding to 0.2 ng of p27), and RT activity of the culture supernatants on day 4 post-infection was measured. In the competition assay for comparison of *in vitro* replication capacity of two kinds of viruses, HSC-F cells infected with individual virus stocks were cocultured to determine which viral genome sequences become dominant in the culture supernatants. HSC-F cells were infected with individual virus stocks (normalized by RT activity) and their coculture started next day. Coculture was continued by transferring the culture supernatant into fresh HSC-F cells every 4 days. RNA was extracted from the coculture supernatant and the Gag CA (capsid)-coding region was sequenced. When only one viral sequence became dominant on day 2 after the coculture initiation, we confirmed that the virus became dominant even in the coculture of the virus-infected cells with larger numbers of the other virus-infected cells in which both viruses were equivalently detected on day 2.

### Analysis of antigen-specific CD8^+^ T-cell responses

We measured antigen-specific CD8^+^ T-cell responses by flow cytometric analysis detecting gamma interferon (IFN-γ) induction after specific stimulation as described previously [41]. Autologous herpesvirus papio-immortalized B-lymphoblastoid cell lines (B-LCLs) were pulsed with individual SIVmac239 epitope-coding peptides (at a final concentration of 1–5 μM) or peptide pools (at a final concentration of 1–2 μM for each peptide) using panels of overlapping peptides spanning the entire SIVmac239 Gag, Pol, Vif, Vpx, Vpr, Tat, Rev, Env, and Nef amino acid sequences (Sigma Aldrich Japan). PBMCs were cocultured with these pulsed B-LCLs under GolgiStop (monensin, BD) presence for 6 hours. Intracellular IFN-γ staining was performed with a CytofixCytoperm kit (BD) and fluorescein isothiocyanate (FITC)-conjugated anti-human CD4 (M-T477, BD), peridinin chlorophyll protein (PerCP)-conjugated anti-human CD8 (SK1, BD), allophycocyanin (APC)-conjugated anti-human CD3 (SP34-2, BD), and phycoerythrin (PE)-conjugated anti-human IFN-γ monoclonal antibodies (4S.B3, Biolegend). Specific T-cell frequencies were calculated by subtracting non-specific IFN-γ^+^ T-cell frequencies from those after antigen-specific stimulation. Specific CD8^+^ T-cell frequencies lower than 0.02% of CD8^+^ T cells were considered negative.

### Statistical analysis

All statistical analyses were performed using Prism software (GraphPad Software, Inc.) with significance set at *p* values of < 0.05. Comparisons were performed by Mann-Whitney U-test or log-rank test.

## Supporting information

S1 Fig Frequencies of dominant nonsynonymous *gag* mutations in plasma viruses obtained from SIV transmitted macaques. Frequencies of the *gag* mutations shown in Fig 3B are shown. Black boxes indicate that the mutation/wild-type ratio was >4/1, while gray boxes indicate that the ratio was more than 1/1 but less than 4/1 in the data obtained by direct sequencing.
(TIF)

S2 Fig Phylogenetic analyses of NGS data on viral Gag CA- and Vif-coding regions in serially SIV transmitted macaques. NGS analyses were performed on SIV Gag CA- and Vif-coding cDNAs obtained from macaque #11 at 3 months (3M) and 1 year (1Y) post-infection and from macaques #21, #22, #31, and #32 at 1 week (1W), 3M, and 1Y post-infection. Phylogenetic analyses of NGS data on viral CA-coding (**A**, **B**) and Vif-coding (**C**, **D**) regions in macaques #11-#21-#31 (**A**, **C**) and #11-#22-#32 (**B**, **D**) are shown. The trees are drawn to scale, with branch lengths measured in the number of substitutions per site.
(TIF)

S3 Fig Phylogenetic analyses of NGS data on viral Nef-coding regions in serially SIV transmitted macaques. Phylogenetic analyses of NGS data on viral Nef-coding regions in macaques #11-#21-#31 (**A**) and #11-#22-#32 (**B**) are shown. The trees are drawn to scale, with branch lengths measured in the number of substitutions per site.
(TIF)

S4 Fig Competition assay for comparison of *in vitro* viral fitness of passaged viruses with wild-type SIV. Comparison between wild-type and PBMC-derived **(A)** or plasma-derived SIVs **(B)**. PBMC-derived or plasma-derived virus-infected cells were cocultured with wild-type virus-infected cells to determine which viruses become dominant by detection of wild-type (Wt) or mutant (Mt) sequences in culture supernatant-derived viral *gag* cDNAs on day 16 (d16) after the coculture start. Representative results (on Gag CA residues at which compared viruses had different sequences) of two experiments on the coculture in which both kinds of viruses were equivalently detected on day 2 (d2) after coculture initiation are shown. In all competitions, the mutant sequences became undetectable on d16, indicating higher *in vitro* viral fitness of the wild-type SIV.
(TIF)

S5 Fig Plasma viral loads in the acute phase after wild-type SIV (WtSIV) and 3pSIV infection. To clearly see the acute phase portion of the data exhibited in Fig 5, changes in viral loads in *90-120-Ia*^+^ (**A**) or *90-120-Ia*^-^ (**B**) macaques up to 3 months after WtSIV (black) or 3pSIV (red) infection are shown.
(TIF)

S6 Fig Comparison of plasma viral loads and survival periods after wild-type SIV and 3pSIV infection in *90-120-Ia*^+^ macaques other than macaque #412. Even the five 3pSIV-infected *90-120-Ia*^+^ macaques other than #412 sharing *89-002-Ip*-derived Mamu-B*007:*02* with macaque #31 showed significantly higher setpoint viral loads (by Mann-Whitney U-test) (**A**) and shorter survival periods (by log-rank test) (**B**) than SIVmac239-infected.
(TIF)
