# Supplementary figures and images for: In vivo virulence of MHC-adapted AIDS virus serially-passaged through MHC-mismatched hosts
Source: PLoS Pathog. 2017 Sep 20;13(9):e1006638. doi: 10.1371/journal.ppat.1006638 (PMC5624644; doi:10.1371/journal.ppat.1006638)

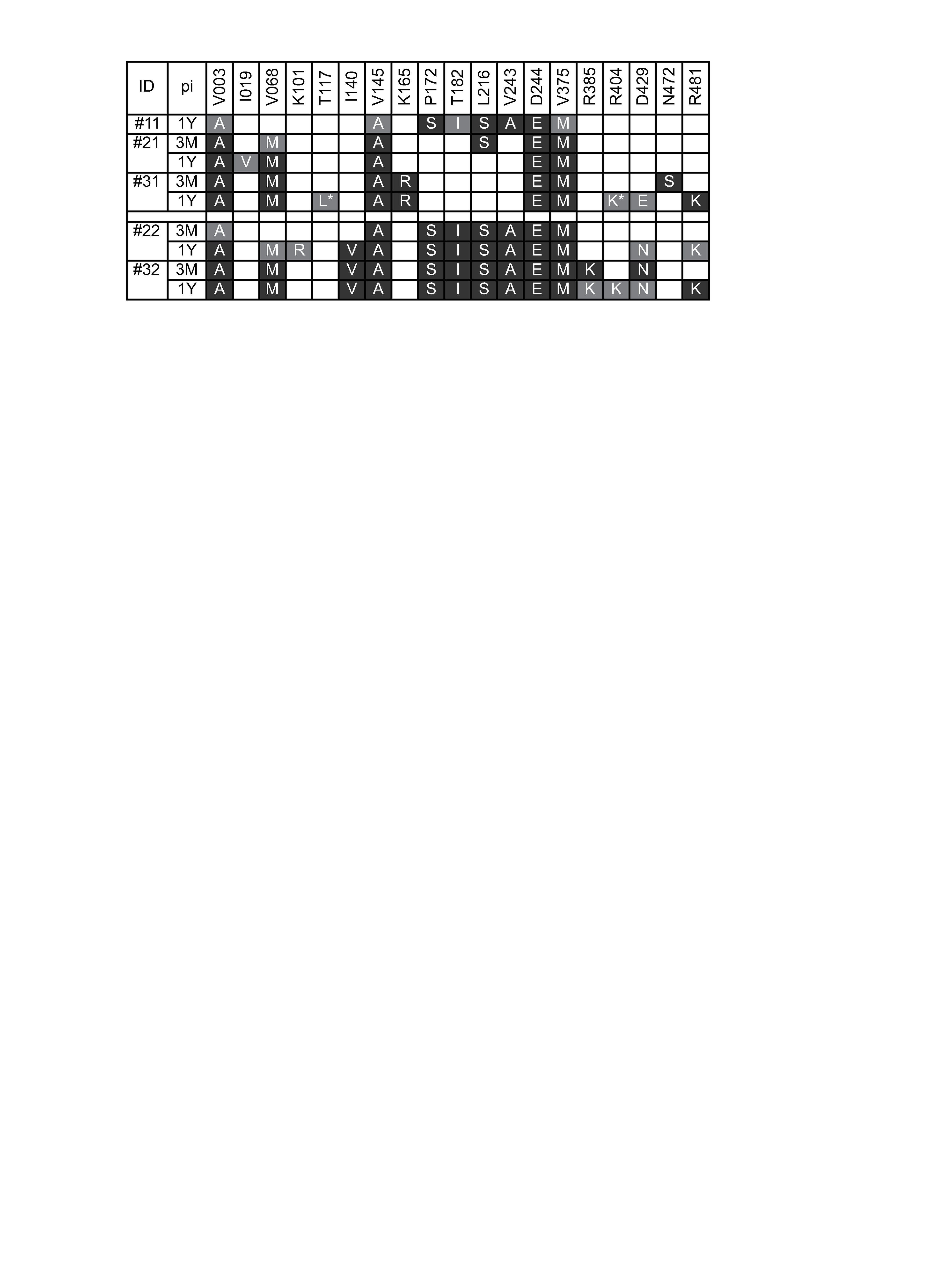

Supplement: S1 Fig — Frequencies of the gag mutations shown in Fig 3B are shown. Black boxes indicate that the mutation/wild-type ratio was >4/1, while gray boxes indicate that the ratio was more than 1/1 but less than 4/1 in the data obtained by direct sequencing. (TIF) [file ppat.1006638.s001.tif]

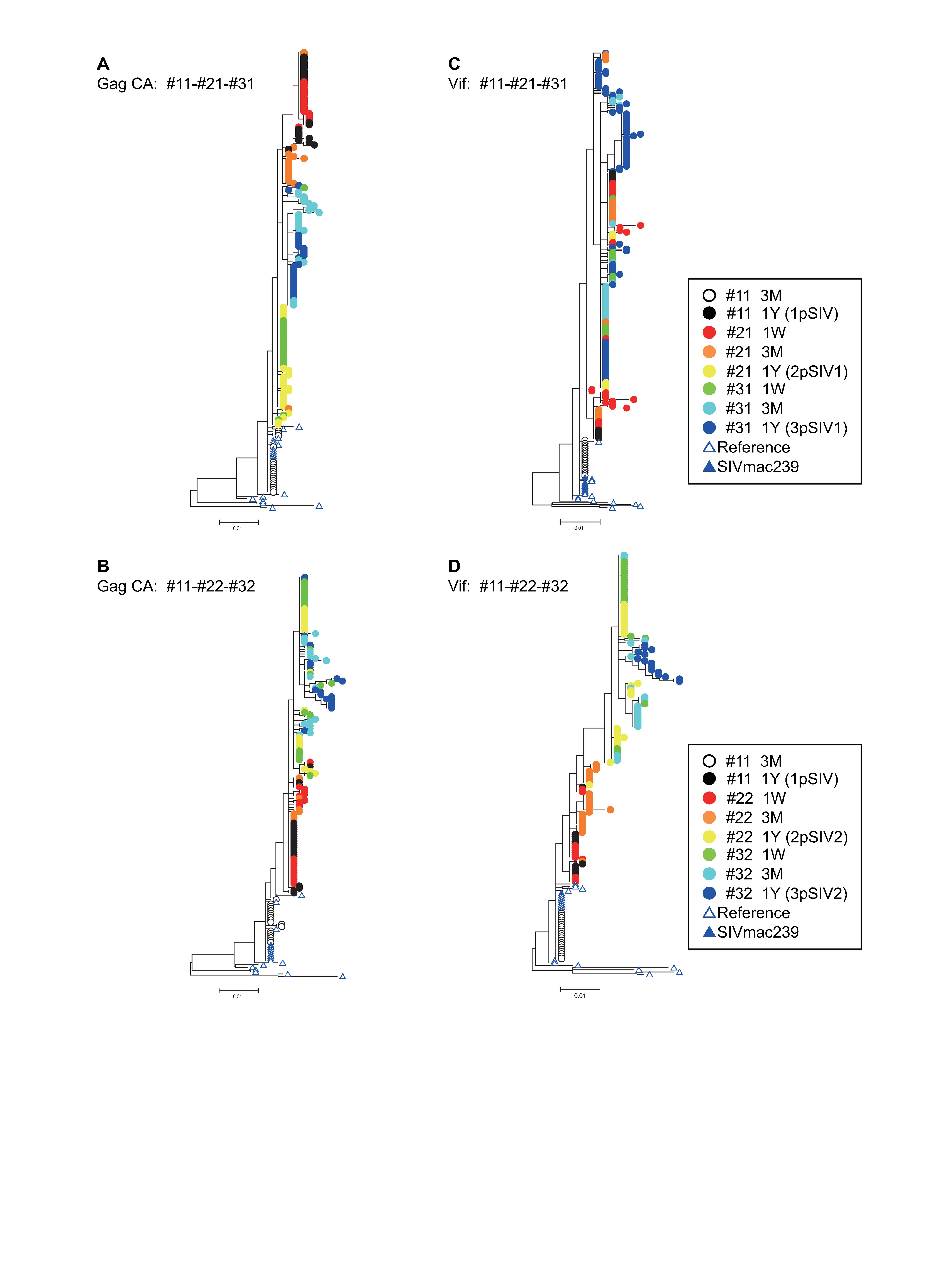

Supplement: S2 Fig — NGS analyses were performed on SIV Gag CA- and Vif-coding cDNAs obtained from macaque #11 at 3 months (3M) and 1 year (1Y) post-infection and from macaques #21, #22, #31, and #32 at 1 week (1W), 3M, and 1Y post-infection. Phylogenetic analyses of NGS data on viral CA-coding (A, B) and Vif-coding (C, D) regions in macaques #11-#21-#31 (A, C) and #11-#22-#32 (B, D) are shown. The trees are drawn to scale, with branch lengths measured in the number of substitutions per site. (TIF) [file ppat.1006638.s002.tif]

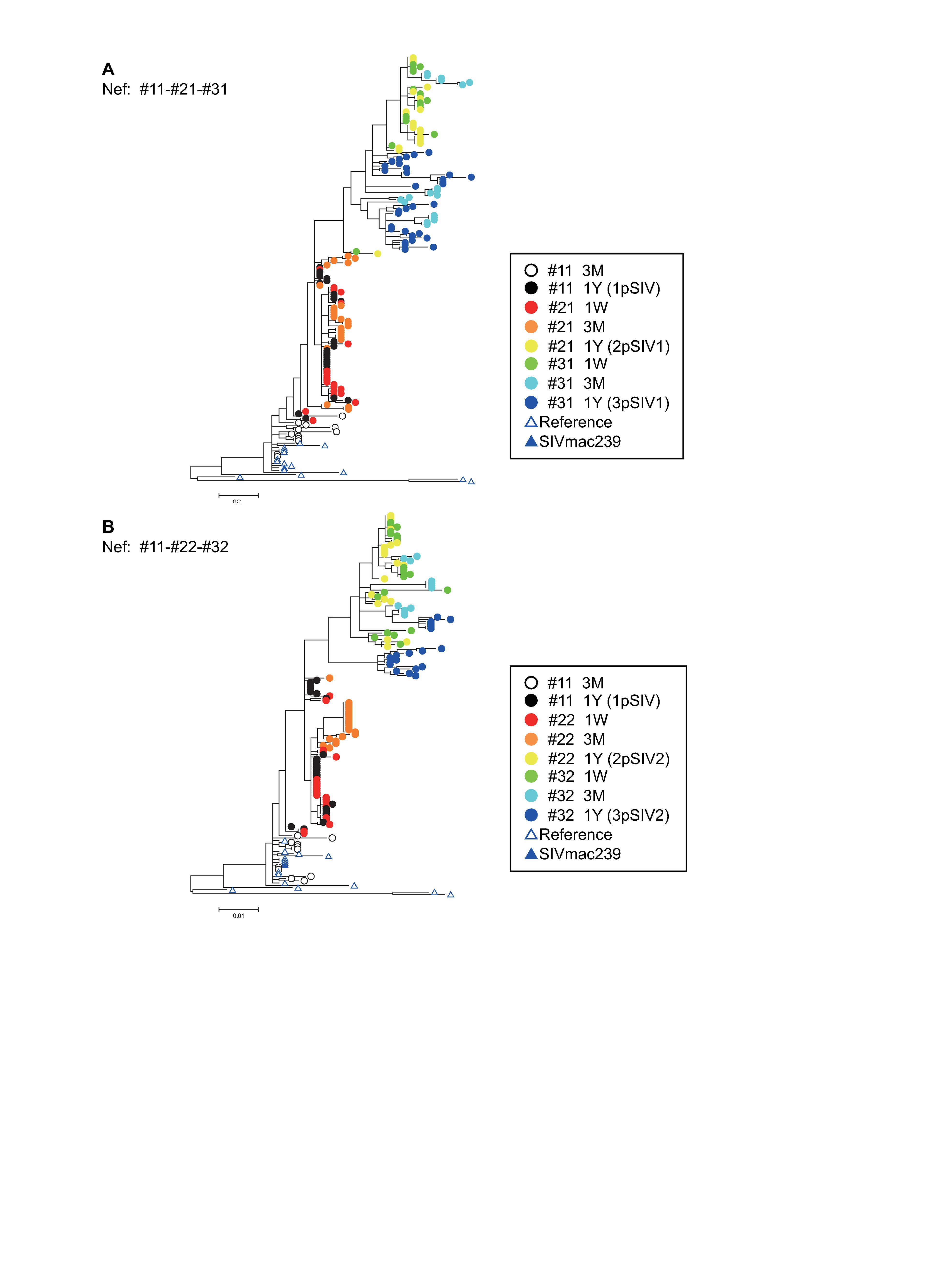

Supplement: S3 Fig — Phylogenetic analyses of NGS data on viral Nef-coding regions in macaques #11-#21-#31 (A) and #11-#22-#32 (B) are shown. The trees are drawn to scale, with branch lengths measured in the number of substitutions per site. (TIF) [file ppat.1006638.s003.tif]

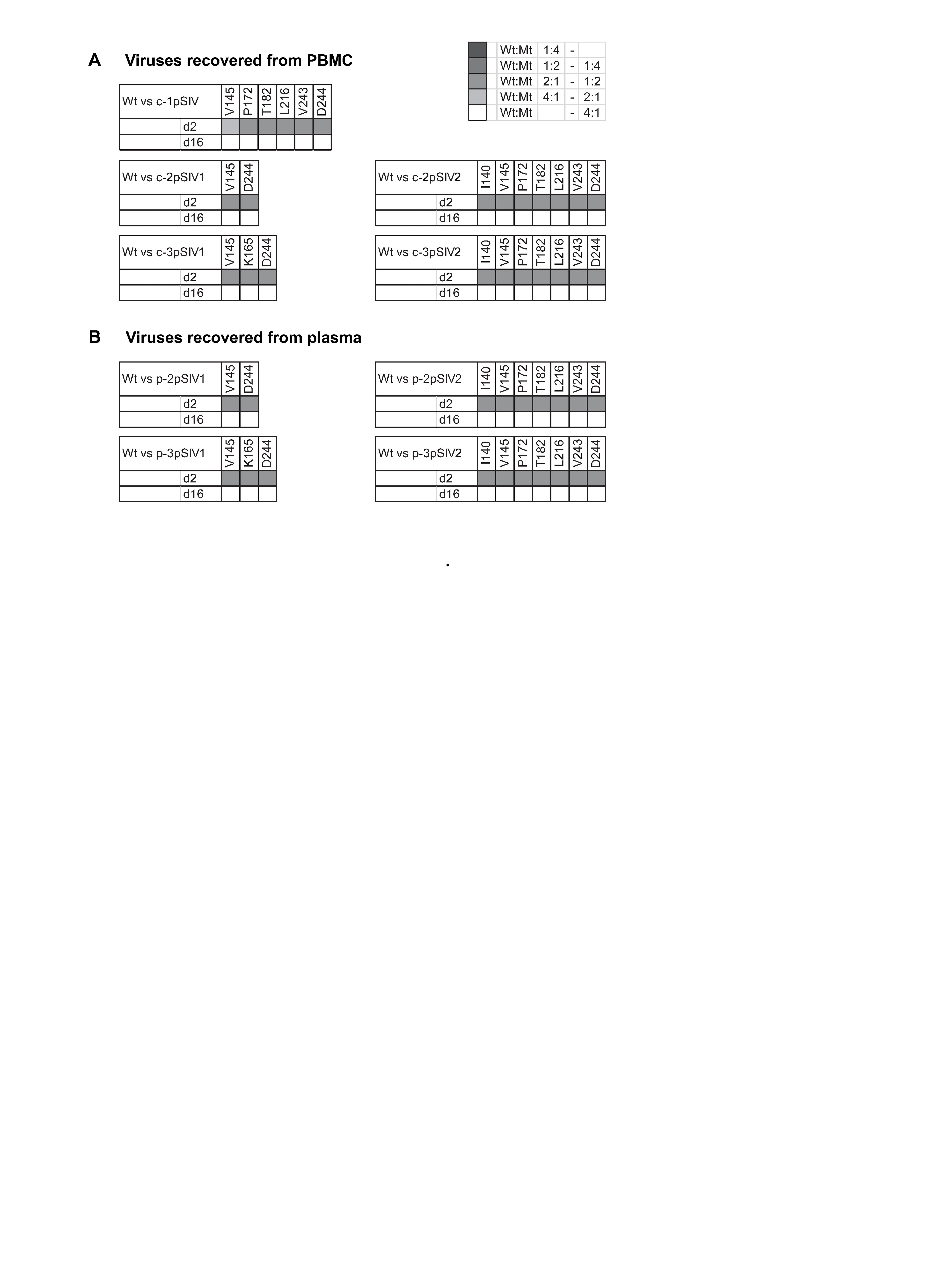

Supplement: S4 Fig — Comparison between wild-type and PBMC-derived (A) or plasma-derived SIVs (B). PBMC-derived or plasma-derived virus-infected cells were cocultured with wild-type virus-infected cells to determine which viruses become dominant by detection of wild-type (Wt) or mutant (Mt) sequences in culture supernatant-derived viral gag cDNAs on day 16 (d16) after the coculture start. Representative results (on Gag CA residues at which compared viruses had different sequences) of two experiments on the coculture in which both kinds of viruses were equivalently detected on day 2 (d2) after coculture initiation are shown. In all competitions, the mutant sequences became undetectable on d16, indicating higher in vitro viral fitness of the wild-type SIV. (TIF) [file ppat.1006638.s004.tif]

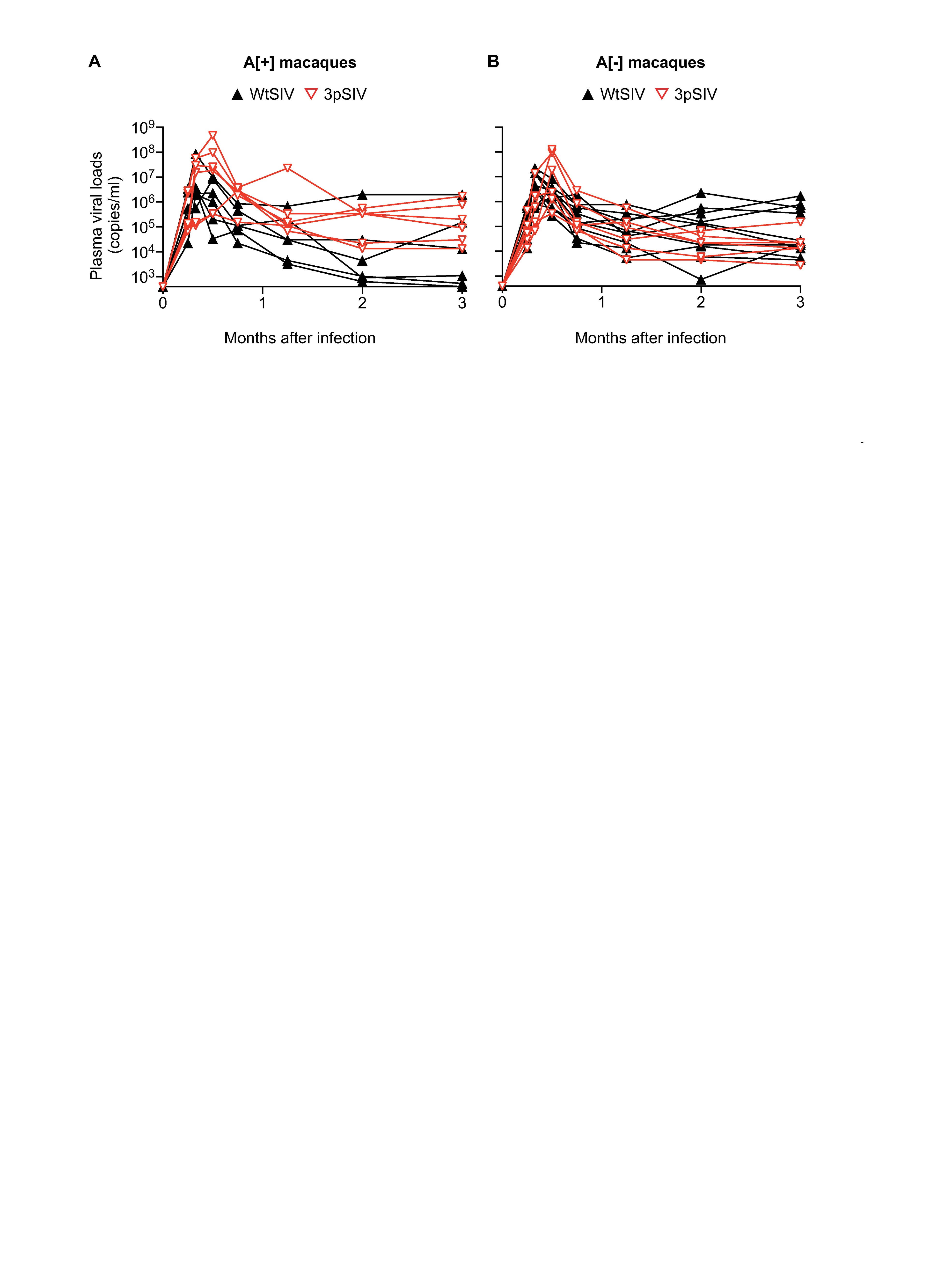

Supplement: S5 Fig — To clearly see the acute phase portion of the data exhibited in Fig 5, changes in viral loads in 90-120-Ia+ (A) or 90-120-Ia- (B) macaques up to 3 months after WtSIV (black) or 3pSIV (red) infection are shown. (TIF) [file ppat.1006638.s005.tif]

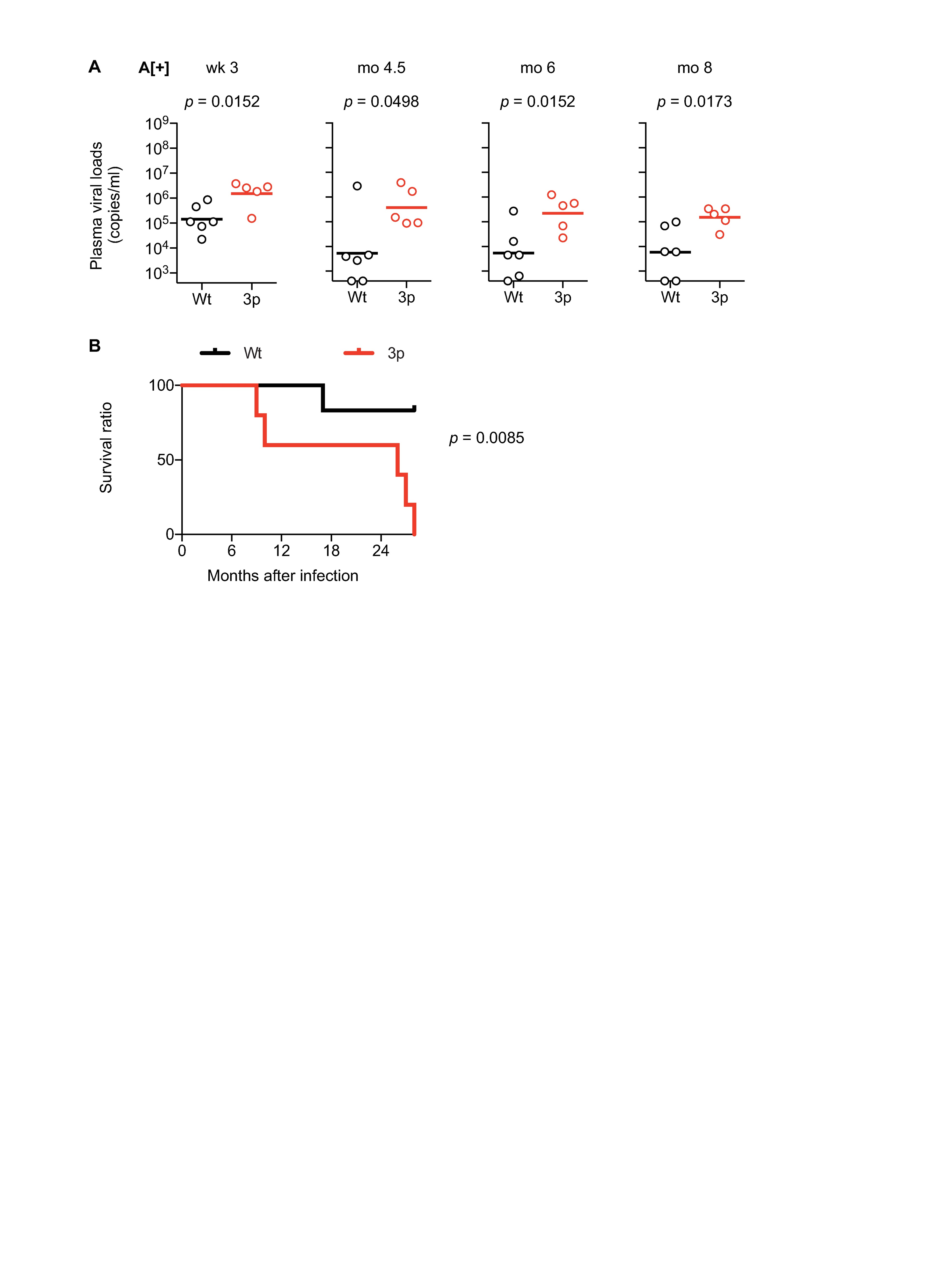

Supplement: S6 Fig — Even the five 3pSIV-infected 90-120-Ia+ macaques other than #412 sharing 89-002-Ip-derived Mamu-B*007:02 with macaque #31 showed significantly higher setpoint viral loads (by Mann-Whitney U-test) (A) and shorter survival periods (by log-rank test) (B) than SIVmac239-infected. (TIF) [file ppat.1006638.s006.tif]
